# Effective Removal of Levofloxacin from Pharmaceutical Wastewater Using Synthesized Zinc Oxid, Graphen Oxid Nanoparticles Compared with their Combination

**DOI:** 10.1038/s41598-020-61742-4

**Published:** 2020-04-03

**Authors:** Christine M. El-Maraghy, Ola M. El-Borady, Omnia A. El-Naem

**Affiliations:** 1 Analytical Chemistry Department, Faculty of Pharmacy, October University for Modern Sciences and Arts (MSA), 11787 6th October City, Cairo, Egypt; 2 Institute of Nanoscience and Nanotechnology, Kafrelsheikh University, Kafrelsheikh, 33516 Egypt

**Keywords:** Analytical chemistry, Environmental chemistry, Environmental sciences, Nanoscience and technology

## Abstract

The presence of antibiotic traces in the aquatic system due to the inefficient treatment of the pharmaceutical wastewater represented threats, such as bioaccumulation and antibiotic-resistance, to the environment and human health. Accordingly, for the first time, the current work utilized the photocatalytic degradation and the adsorption approach for Levofloxacin (LEVO) in pharmaceutical wastewater using new designed nano aspects. Therefore, spherical Zinc oxide nanoparticles (ZnONP) sized 17 nm and ultrathin sheet-like structure graphene oxide nanosheets (GONS) with layer thickness ~5 nm were fabricated separately or in a combination between them then characterized via Transmission Electron Microscope (TEM), Scanning Electron Microscope (SEM), X-Ray Diffraction (XRD), Fourier Transforms Infrared Spectroscopy (FTIR), absorption spectra (UV-Vis) and Brunauer-Emmett-Teller (BET). Additionally, several parameters were investigated to evaluate the potential of the removal process, such as pH, the exposure time to UV radiation, the type and concentration of the nanoparticles (NPs) and the initial concentration of the drug using a mixed fractional factorial design. The most effective parameter for LEVO removal was the NPs type followed by the initial drug concentration. Furthermore, an RP-HPLC/UV method was developed and validated for measuring the percentage of removal for LEVO drug. The highest percentage removal for both 50 and 400 µg mL^−1^ LEVO was 99.2% and 99.6%, respectively, which was achieved using ZnONP/GONS combination at pH 9 ± 0.05 and UV light exposure time 120 min. In addition, the negative antibacterial activity of the treated wastewater sample confirmed the drug removal. The established protocol was successfully applied on wastewater samples collected from a pharmaceutical company that encouraged researchers to mainstream this design to be applied on other pharmaceutical wastewater drugs.

## Introduction

**Figure 1:**
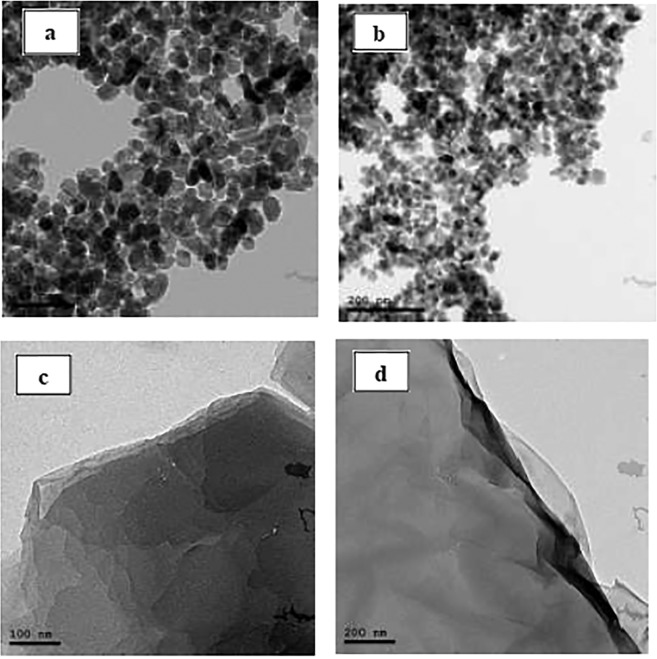
TEM image of ZnONP taken at different scale (**a**) 100 nm and (**b**) 200 nm and GONS at (**c**) 100 nm and (**d**) 200 nm.

The environmental and human risks due to the occurrence of antibiotics in the aquatic environment, even in low concentration, have attracted considerable concern. Antibiotics have been detected worldwide in the aquatic environment, indicating their inefficient removal from wastewater utilizing the conventional methods of water treatment^1^. The presence of antibiotics in the ordinary water comes mainly from the discharges of sewage treatment plants (STPs) and pharmaceutical manufacturing plants^2–5^. The presence of antibiotic traces in the aquatic system can cause bio-accumulation and resistance in bacterial populations, making them ineffective for treatment in the near future^6^. Therefore, there is a serious need to merely develop a simple, effective and low-cost method for removing of such contaminants. In this current study, the LEVO (Fig. 1, supplemental materials) was chosen as an example of antibacterial fluoroquinolones (FQs) and one of the essential medicines listed by the World Health Organization’s (WHO)^7^. Their presence in the aquatic environment can cause development of antibiotic-resistant bacteria^6^ and genotoxicity^8^. In addition, FQs are prescribed largely in human and veterinary uses and are partially metabolized in the body, so a significant amount of the drug is excreted in the same form which definitely affects the aquatic ecosystem^9^. There are many techniques used for wastewater treatment and remediation like conventional methods such as biological processes, coagulation/flocculation/sedimentation^10–12^ and by sand filtration or ozonation^13,14^ which are not designed to remove highly polar contaminants as the antibiotics. There are non-conventional methods such as oxidation, adsorption, reverse osmosis, ion exchange and combined methods^15^. Two techniques were demonstrated for an efficient removal of the antibiotic from wastewater: the advanced oxidation process (AOPs) and the adsorption technique. The AOPs depends on the photocatalytic degradation of organic compounds and has the advantage of being environment friendly as it converts the organic pollutants into CO_2_ and H_2_O^16^. The adsorption technique is commonly used to describe the tendency of the molecules in fluid phase to adhere to a solid surface^15^ and has the advantage of simplicity of design and operation and is relatively inexpensive^2^. The literature survey revealed several photocatalytic degradations of LEVO using different photocatalysts such as the TiO_2_ with drug degradation not more than 90%^17–19^, Bi_2_WO_6_ nanocuboids with approximately 80% degradation in 150 min^20^, Ag/AgBr/BiOBr microplates under visible light with degradation about 74% under 90 min of irradiation^21^ and a combination of Ag_2_O/TiO_2_ quantum dots under visible light with 81% of LEVO degradation in 90 min^22^. There was a reported method for the removal of FQs using the magnetite pectin as adsorbent^23^. The need for a simple, effective and low-cost method entered the line with the emergence of a new science: the nanotechnology which has entered its applications in many fields. The NPs are considered potent adsorbents, disinfectants and catalysts and they are also used as sensors due to their high specific surface area expressed to large reactivity^24^. In this work, two types of metal oxide nanoparticles were employed: ZnONP and GONS. ZnONP was used as an efficient alternative disinfectant for wastewater system because of its catalytic activity^25^ and it has a small particle size (hence high surface area) which aids in its efficiency^26^. ZnONP of concentration 0.34–1.42 μg L^−1^ was used in treated wastewater in Europe in 2008^27^ and it was increased to 1.7–21 μg L^−1^ in 2014^28^. In recent years, GONS, a heavily oxygenated graphene derivative, has been extremely explored for biomedical applications as well as in membranes for water treatment^29,30^. GO has a high surface area with a large number of polar oxygen-containing functional groups^31^, which makes it a valuable adsorbent to be used in removal of organic compounds. The aim of the present work was to develop an efficient simple method for pharmaceutical wastewater treatment containing LEVO, as a model of antibacterial FQS in wastewater, and to achieve higher removal for the LEVO than the other published works. A mixed fractional factorial design was carried out to optimize the removal process and to study the effect of various factors affecting the LEVO removal. A protocol using RP-HPLC/UV has been developed and validated for monitoring of LEVO throughout the work of treatment. The optimum set of conditions for the highest removal percentage was applied on pharmaceutical wastewater samples. The introduction of such a protocol for the treatment of pharmaceutical wastewater will help to improve human and ecological health.

## Experimental

### Chemicals and samples

Standard LEVO was obtained from Sanofi pharmaceutical company (Egypt). For the preparation of NPs: zinc acetate dihydrate, sodium hydroxide, sulfuric acid (95%), sodium nitrate (99.9%), and potassium permanganate (99.9%, KMnO_4_) were purchased from sigma Aldrich; Graphite (99.99%) and hydrogen peroxide (H_2_O_2_ 36%) from Alfa Aesar; Methanol HPLC grade (99.8%) from LobaChemie PVT.LTD, India; and Potassium dihydrogen phosphate and ortho-phosphoric acid from Sharlau, Spain. For the antibacterial activity test: saline solution, barium chloride, sulphuric acid (ADWIC company, Egypt) and Mueller-Hinton agar were used.

### Instruments

Morphology information including the particle size and shape of fabricated NPs were measured using the Transmission Electron Microscopy (HR-TEM), JOEL JEM-2010 operating at an accelerating voltage of 200 kV attached with Gatan digital camera Erlangshen ES500. Structural composition was achieved by FT-IR measurements via JASCO spectrometer; samples were scanned from 4000–400 cm^−1^. The crystallography of the synthesized NPs powder and patterns of X-Ray Diffraction were analyzed using SHIMADZU XRD 6000 diffractometer with CuKa1 radiation (k = 1.54056A°) operating at voltage and current of 40.0 (kV) and 30.0 (mA), respectively. The pore characteristics such as average pore diameter and specific surface area of the NPs were determined according to the Brunauer-Emmett-Teller (BET) and Barrett-Joyner-Halenda (BJH) methods. HPLC Agilent 1200 series, with a multiple wavelength detector, microvacuum degasser and thermostatic column compartment, was used for measuring the LEVO concentration throughout the work of treatment. ChemStation software (Agilent Technologies, Germany) was used for displaying the results. Phenomenex C_18_ column (150 mm × 4.6 mm, 5 μm particle size) was obtained from Agilent Technologies, Polo Alto, CA, USA, and the pH-meter was Jenway3505, UK. The experimental design was performed using design Expert Software version 7.0 (Stat-Ease Inc., Statistics made easy, Minneapolis, USA). Shimadzu UV-visible 1800 spectrophotometer, Japan, was connected to UV-Probe 2.32 software. The incubator was used for the antibacterial activity test (Jouan, USA).

### Synthesis of Zinc oxide nanoparticles (ZnONP)

ZnONP has been synthesized according to Beek *et al*. method^32^ and Weller *et al*.^33^ through the hydrolysis method of zinc acetate dihydrate by KOH at low temperature not more than 70 °C in alcoholic medium (solvothermal method), but with some slight modification. Firstly, 3.35 mmoL of Zinc acetate dihydrate was dissolved in 32 mL methanol and mixed very well to obtain a homogenous solution using magnetic stir; another solution of 6.60 mmoL NaOH was prepared in methanol. Then, NaOH solution was added dropwise to the zinc acetate solution while carefully adjusting the temperature solution at 60 °C with vigorous stirring. The solution became turbid with a white precipitate upon addition of the hydroxide indicating the formation of ZnONP. After the addition of NaOH was completed, the solution mixture was left to be stirred for 120 min while the heater is off. Finally, the precipitate formed was left to settle down and was washed four times with methanol then dried in the oven at 100 °C.

### Synthesis of graphene oxide nanosheets (GONS)

GONS was synthesized via Improved Hummer’s method^34^. In this method, the graphite was used as the precursor for the production of graphene while a strong oxidation of the graphite was done leading to a weak π-π stacking between the graphite layers; herein, several oxygenated functional groups are inserted onto the surface of GO. This is followed by a disruption for the sp^2^ graphite structure which makes it possess many intrinsic characteristics such as electrical and mechanical properties^35,36^. Briefly, a solution of sulfuric acid (25 mL) containing 0.5 g of graphite was prepared, followed by adding 0.5 g of NaNO_3_ with moderate stirring (20 min). After that, the mixture solution was kept at 10 °C in an ice bath. When reaching the desired temperature, 3 g of KMnO_4_ was gradually added over 15 min and the solution was kept in a temperature of not more than 10 °C; this will enhance the homogeneity of the formed GO nanosheets. The reaction solution was left to be stirred at 35 °C overnight. At that time, 50 mL of deionized water was added dropwise. While addition, a remarkable increase in the temperature was observed; therefore, we adjusted it so as not to exceed 90 °C. After that, the solution was left to be stirred at the same temperature for 60 min. Finally, 140 mL of warmed deionized water was slowly added to the solution followed by addition of 5 mL of H_2_O_2_; the solution color turned into brownish yellow indicting the formation of GO. The decantation was done for the mother liquor, then the brown precipitate was washed several times by distilled water to remove any unreacted substances, and then it was filtered and dried in the oven at 60 °C.

### Preliminary studies

In the present investigation, standards of LEVO (100 μg mL^−1^) were prepared in distilled water. Studies were performed to assess the effect of presence and absence of photocatalyst with UV exposure. The LEVO standard solution was carried in a UV chamber and exposed to a UV lamp (254 nm, 1012 μW cm^−2^) at variable durations (60 min or 120 min) in the presence or absence of the photocatalytic NPs (ZnONP or GONS or combination of both) and at 2 pH units (7 and 9). After the incubation time, the samples were analyzed using UV-Vis spectrophotometer.

### Experimental design

The mixed fractional factorial design (2^4^ × 3^1^) was used to evaluate the effect of four factors; the pH, the exposure time to UV radiation, the type and concentration of the NPs and the initial concentration of LEVO drug. Two levels for each factor were chosen: low level (−1) and high level (+). Moreover, for the type of the NPs, three levels were assessed (ZnONP, GONS and a combination of both) as shown in Table 1. Forty-eight samples were prepared with various levels of the factors to choose the optimum conditions for the highest removal of LEVO drug, as shown in Table 2. The samples were analysed after filtration using RP-HPLC/UV. The removal percentage was calculated as follows:

$$ \% {\rm{removal}}=({\rm{C}}\mbox{'}-{\rm{C}})/{\rm{C}}\mbox{'}\times 100,$$

where C’ is the concentration of intact drug solution and C is the concentration of the treated drug solution.

**Table 1:** The factors and their levels used for the mixed fractional factorial design experiment.

Factor name	Low level (−1)	High level (1)
pH	7	9
Time of UV exposure	60 min	120 min
Drug Conc.	50 ug mL^−1^	400 ug mL^−1^
ZnO	0.4 g L^−1^	1 g L^−1^
GO	0.4 g L^−1^	1 g L^−1^
ZnO: GO	0.4:1 g L^−1^	1:0.4 g L^−1^

**Table 2:** Design matrix for the mixed fractional factorial (2^4^ × 3^1^) employed for LEVO photocatalytic degradation and results of the RP-HPLC.

Run no	pH	time	Levo comc.	NP conc	NP type	Average peak area	Degradation %
1	−1	1	−1	−1	ZnO:GO	7	99.34%
2	1	1	1	−1	ZnO	1423	29.87%
3	−1	−1	1	1	ZnO:GO	50	97.54%
4	1	−1	1	−1	GO	225.9	88.87%
5	1	1	1	1	ZnO	1348.8042	33.52%
6	1	1	1	1	GO	217	89.31%
7	1	1	−1	1	ZnO:GO	22	97.92%
8	1	−1	−1	−1	ZnO	743	29.77%
9	1	−1	−1	−1	ZnO:GO	28	97.35%
10	−1	−1	1	−1	ZnO:GO	70	96.55%
11	−1	−1	−1	1	GO	86	91.87%
12	−1	1	1	−1	GO	222	89.06%
13	−1	−1	−1	−1	ZnO:GO	25	97.64%
14	−1	1	−1	−1	GO	112.03658	89.41%
15	−1	−1	1	−1	GO	255	87.43%
16	−1	−1	−1	1	ZnO:GO	30	97.16%
17	−1	−1	1	1	ZnO	1411.43237	30.44%
18	−1	−1	−1	−1	GO	112.95795	89.32%
19	1	1	−1	−1	GO	109.23647	89.68%
20	−1	−1	1	−1	ZnO	1440.86401	28.99%
21	1	−1	1	1	GO	188	90.73%
22	1	−1	1	1	ZnO	1431.43237	29.45%
23	−1	−1	−1	−1	ZnO	752	28.92%
24	−1	1	1	1	ZnO:GO	44.5	97.81%
25	−1	−1	−1	1	ZnO	732	30.81%
26	−1	1	1	−1	ZnO	1400	31.00%
27	1	−1	−1	−1	GO	133.50594	87.38%
28	−1	1	−1	1	ZnO	711	32.80%
**29**	**1**	**1**	**1**	**−1**	**ZnO:GO**	**16.01124**	**99.21%**
30	1	−1	−1	1	ZnO:GO	40	96.22%
31	−1	1	1	−1	ZnO:GO	20	99.01%
32	1	−1	1	1	ZnO:GO	66	96.75%
33	1	1	1	1	ZnO:GO	50	97.54%
34	−1	1	1	1	GO	165	91.87%
**35**	**1**	**1**	**−1**	**−1**	**ZnO:GO**	**3.93124**	**99.63%**
36	−1	1	−1	1	GO	126	88.09%
37	1	−1	−1	1	ZnO	747	29.40%
38	−1	1	1	1	ZnO	1348.6	33.53%
39	1	−1	1	−1	ZnO:GO	77	96.21%
40	1	1	−1	1	GO	128	87.90%
41	−1	1	−1	1	ZnO:GO	28	97.35%
42	1	−1	1	−1	ZnO	1354	33.27%
43	−1	1	−1	−1	ZnO	712	32.70%
44	1	1	1	−1	GO	210	89.65%
45	1	1	-1	1	ZnO	697	34.12%
46	1	-1	-1	1	GO	94.3	91.09%
47	1	1	-1	-1	ZnO	700.3	33.81%
48	-1	-1	1	1	GO	191	90.59%

### Chromatographic conditions

The optimum result was obtained using Phenomenex C_18_ (150 mm × 4.6 mm, 5 μm particle size i.d.) and mobile phase consisting of methanol: phosphate buffer (pH 6) in a ratio 50:50 v/v. The pH of the buffer was adjusted using orthophosphoric acid. Isocratic elution was employed at flow rate 1 mL min^−1^ and the UV detection at 294 nm. The samples were filtered before injection using syringe filter paper (0.45 μm, PTFE).

### Construction of calibration curve and validation

The calibration curve was linear in the concentration range (10–500 μg mL^−1^). The obtained regression equation (y = 25.023x– 22.852) was used to calculate the concentration of LEVO residual after each degradation trial. The validation procedure was done according to the ICH guidelines^37^ for determination of linearity, accuracy, precision, specificity, limit of detection (LOD) and limit of quantification (LOQ).

### Application to pharmaceutical wastewater

The optimum conditions for degradation of LEVO were applied on incurrent samples of pharmaceutical wastewater. The wastewater was obtained after three cycles from the cleaning production lines of one batch of LEVO. According to the manufacturer’s protocol, the cleaning procedure was performed using boiling distilled water for three successive cycles. Samples were collected after each washing cycle and pooled together; their pH was measured and then they were stored at −20 °C. The concentration of LEVO in the three collected samples was measured three times each using RP-HPLC/UV and the pH was adjusted to nine, then the samples were exposed to UV radiation (254 nm, 1012 μW cm^−2^) for 120 min in the presence of a combination of ZnONP:GONS (0.4:1 g L^−1^). At the end of the exposure time, the samples were filtered and injected into the HPLC system as mentioned previously.

### Antibacterial activity test using agar diffusion method

To confirm the effective removal of LEVO and the lack of its antimicrobial activity, the agar diffusion method was done on the treated water sample achieving the optimized conditions compared with the untreated one^38^. In a typical experiment, a 100 μL of fresh culture of *Escherichia coli* ATCC 29522 (approximately 10^8^ CFU mL^−1^) was uniformly spread onto Mueller-Hinton agar plates using a sterile swab. The inoculated plates were allowed to dry at room temperature for 20 min using a sterilized cup-borer, and two wells were made in the agar. The treated and untreated water samples (100 μL) were dispensed into the wells. The Plates were incubated at 37 °C for 18 hours. After the incubation period, the diameter of the zone of inhibition for each sample was measured.

## Results and discussion

### Synthesis and characterization of ZnONP and GONS

#### The transmission electron microscope (TEM)

The morphological essential information for the prepared NPs was examined via the TEM imaging. The TEM images obtained for a suspended solution of ZnONP represented in Fig. 1a,b showed a high uniform size of elongated spherical-shaped NPs that possessed an average size of 17 nm. On the other hand, in the present context, the GONS was produced by oxidation of graphite using modified Hummers method, leading to a combination between KMnO_4_ and H_2_SO_4_ producing diamanganese heptoxide (Mn_2_O_7_), an active oxidant that oxidizes the graphite-unsaturated double bonds of the formed graphene oxide^39^. The TEM image for GONS shown in Fig. 1c,d exhibited an ultrathin sheet-like structure, with a smooth surface, while several folds and wrinkles were observed especially at the edges. In addition, the TEM result sufficiently indicates two-dimensional nanosheets produced for GO with approximately 6 μm in size and with layer thickness of about 5 nm.

### The scanning electron microscope (SEM)

**Figure 2:**
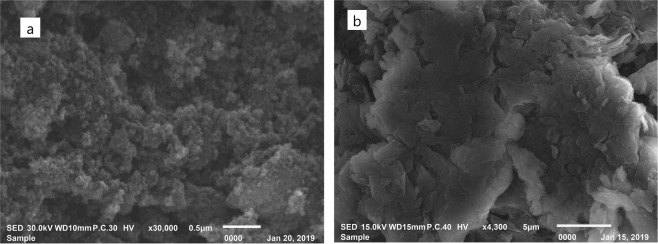
The SEM micrograph image of the synthesized (**a**) ZnONP and (**b**) GONS.

The morphology examination and the surface characterization of the synthesized NPs were also observed via SEM. A micrograph in Fig. 2a presented the SEM image of ZnONP, while its size was predicted to be around 17 nm, having a spherical shape with high order and arrangement. Furthermore, the SEM obtained for GONS depicted in Fig. 2b showed that GONS consists of crumpled thin sheets with some random aggregation. Moreover, clear folds on the surface of GONS were observed. The two-dimensional structure of GONS was a characteristic feature in the SEM image too.

### The BET

**Figure 3:**
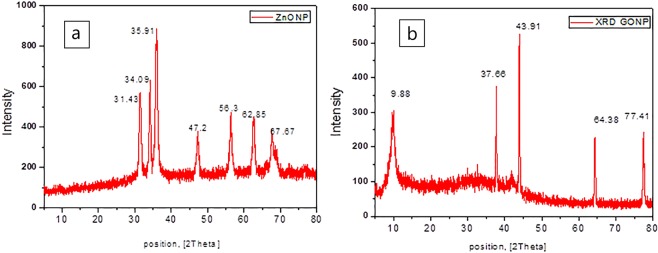
XRD pattern of (**a**) ZnONP and (**b**) GONS.

The BET analysis was performed for the nano samples as an important tool of analysis to prove that the high catalyst active surface was ready for adsorption. The obtained isotherm (Figs. 2 and 3 supplemental materials) indicated that the specific surface area of synthesized ZnONP and GONS were 27 m^2^ g^−1^ and 53 m^2^ g^−1^, respectively. The results revealed that the adsorption capacity of GONS will be superior to ZnONP due to its higher surface area. In addition, the presence of a hysteresis loop in isotherm of GONS confirms the formation of porosity over its surface that may lead it to be a highly effective material towards adsorption of the drug.

### The X-ray diffraction (XRD)

The crystallinity and the structure of the produced NPs were confirmed by subjecting the samples to the XRD analysis, while the magnitude and location of peaks will provide those relevant information. The XRD pattern of ZnONP (Fig. 3a) showed three peaks with 2θ at 31.43, 34.09 and 35.91 that may be corresponding to (100), (002) and (101), respectively. The position of those peaks revealed the presence of ZnONP in a hexagonal wurtzite structure. As reported in earlier studies, the minor boarding for XRD peaks at (100), (002) and (101) may be ascribed to the formation of spherical rather than rod nanoparticles^40^. The other three peaks at scattering angles 2θ of 47.2, 56.3 and 62.85 may be corresponding to (102), (110) and (200) crystal plane, respectively.

The XRD pattern for GONS (Fig. 3b) showed weak and sharp reflection peak at 2θ of 9.88° attributed to the (001) reflection plan for GONS with layer-to-layer distance (d-spacing) within 8.8 A° compared with that appearing for graphite only at 3.4 A° and this increasing in d-spacing was illustrated by Yan *et al*.^41^, and they attributed it to the introduction of hydroxyls and carboxyls functional groups during the oxidation onto the surface of used graphite confirming formation of GONS.

### Fourier transforms infrared spectroscopy (FT-IR)

**Figure 4:**
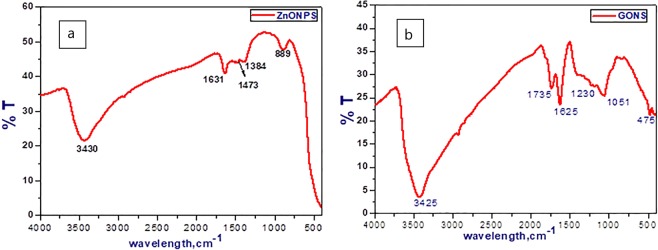
FT-IR chart for (**a**) ZnONP and (**b**) GONS.

FT-IR measurements were done to verify the bond structure of nanoparticles. Figure 4a shows the infrared absorption spectrum of ZnONP scanned in the range from 4000–400 cm^−1^. The spectrum exhibited the appearance of a band centered at 3430 cm^−1^ due to the O-H vibration mode that may present from the alcohol adsorbed on the ZnONP surface. Moreover, two weak and broad bands due to the symmetric stretching of C-O. C-O-C groups were located at 1473 and 1384 cm^−1^, respectively. Also, the peaks at 1631 and 889 cm^−1^ can be assigned to Zn-O stretching and deformation vibration mode, respectively, whereas the peaks that appear below 1000 cm^−1^ in the metal oxides IR chart are considered as fingerprints for existence of metal oxide. All the above frequencies observed in the IR chart of ZnONP were in accordance with the results obtained previously by H. Kumar and R. Rani^42^. Moreover, the GONS FT-IR spectrum in Fig. 4b exhibited a peak around 3425 cm^−1^ due to the stretching vibrations of the O-H group and another two attached sharp peaks were observed at 1735 cm^−1^ and 1625 cm^−1^; those can be attributed to stretching vibration of C=O present in carboxylic and/or carbonyl moiety groups. Furthermore, the two peaks achieved at 1230 and 1051 cm^−1^ are assigned to the C–O stretching vibrations^43^.

### The absorption spectra (UV-Vis) measurements

**Figure 5:**
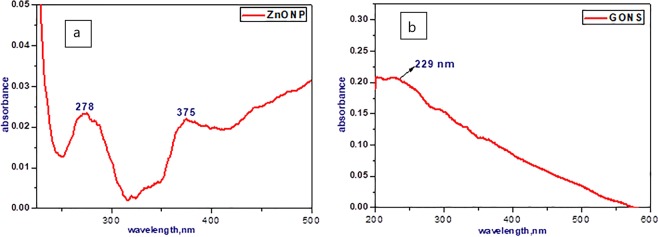
UV-Vis absorption spectrum of (**a**) ZnONP and (**b**) GONS.

The optical properties of the ZnONP and GONS were studied by measuring their absorption spectra using UV-Vis spectrophotometer. The absorption spectrum of ZnONP (Fig. 5a) exhibited an excitonic absorption peak at 277 nm accomplished with another peak around 357 nm which suggested that the synthesized ZnONP were with high purity and crystallinity; this result was matched with Oladiran *et al*.^44^. However, the UV-Vis absorption spectrum of a suspended solution of the GONS (Fig. 5b) revealed the appearance of a main peak located at 229 nm, corresponding to the π → π * transitions for aromatic C–C bond, and a shoulder appeared at about 302 nm that can be arising to the π → π * transitions of C=O bonds on the surface of GO^45^. The UV-Vis measurements and the FT-IR results confirm the existence of oxygen functional groups onto GONS.

### Preliminary studies for LEVO removal

**Figure 6:**
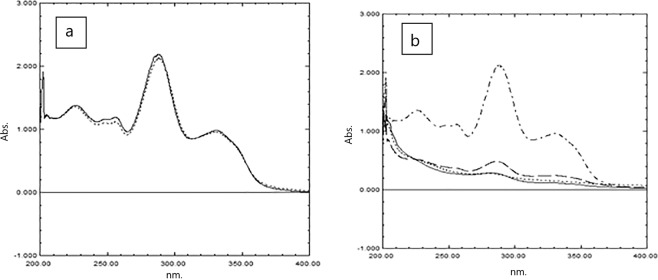
(**a**) Absorption spectra of LEVO (100 μg mL^−1^) before (……) and after (___) exposure to UV radiation for 60 min and (**b**) Absorption spectra of LEVO (100 μg mL^−1^) (.-.-.) in presence of ZnONP (–), GONP (….) and a combination of (___) after exposure to UV radiation for 60 min.

The preliminary investigation for LEVO removal was evaluated by measuring the absorption spectra of four standard solutions of LEVO (100 μg mL^−1^) at its λ max (287.5 nm); one solution acted as a control free from NPs, another two were treated with ZnONP and GONS separately, and the fourth one was treated with mixed NPs. After that, the solutions were exposed to UV radiation for 60 min. After the exposure time was finished, the absorption was measured again, while a very small decrease in the absorption was observed for the control sample (Fig. 6a). On the other hand, a significant decrease in the absorption spectra of LEVO was detected after the exposure to UV radiation in the three water samples treated with NPs (Fig. 6b).

### Experimental design

The mixed fractional factorial design (2^4^ × 3^1^) was employed to study the significance of each chosen factor and their interactions and to optimize the conditions for maximum removal of LEVO from wastewater as listed in Table 1. Furthermore, the forty-eight samples were analysed by RP-HPLC/UV under variable conditions (Table 2). The optimum conditions for removal of LEVO were noticed at run 35 and 29 as shown in Table 2 at pH 9 ± 0.05 after exposure to UV radiation for 120 min in the presence of a combination of ZnO:GO (0.4:1 g L^−1^) for both drug concentrations 50 and 400 µg mL^−1^ as initial concentrations. The results indicated that the optimized condition mention above is independent of the drug concentrations. The removal of the drug was effective although an extremely small amount of the NPs can be utilized which was also very beneficial economically.

### RP-HPLC/UV analysis method

**Figure 7:**
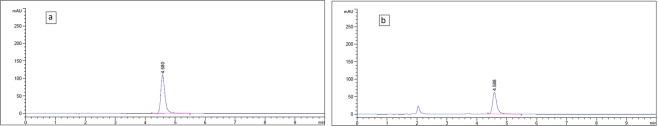
HPLC chromatogram of intact standard LEVO (50 μg mL^−1^) (**a**) before treatment and (**b**) after degradation using 1 g L^−1^ ZnO NP.

Different trials were done to obtain a well symmetric peak of the studied drug LEVO, using different mobile phase compositions and different columns (C_8_ and C_18_). A good resolution was obtained over 4.5 min using Phenomenex C_18_ column (150 mm × 4.6 mm, 5 μm particle size i.d.) and mobile phase consisting of methanol: phosphate buffer (pH 6) in a ratio of 50:50 v/v. Isocratic elution was employed at flow rate 1 mL min^−1^ and the UV detection at 294 nm. Figure 7a represented the chromatogram of intact LEVO before treatment and Fig. 7b represented it after treatment using 1 g L^−1^ ZnONP. We can observe the great decrease in peak area of LEVO which proved its degradation, while no additional peak was detected which indicated that the drug was degraded to H_2_O and CO_2_ according to the reported mechanism^46^. The calibration curve for LEVO was constructed showing a linear relationship over the concentration range of 10–500 μg mL^−1^. Regression validation parameters and system suitability parameters were calculated according to ICH guidelines summarized in Table 3 ^37^.

**Table 3:** Summary of system suitability and validation parameters for the proposed HPLC/UV method.

Parameters		HPLC	Reference values^*^
Linearity (µg mL^−1^)		10–500	
Correlation coefficient (r)		0.9997	
Slope		25.023	
Intercept		22.832	
Standard deviation of residuals from line		23.58	
LOD (µg mL^−1^)		3.109	
LOQ (µg mL^−1^)		9.423	
Accuracy (Recovery % ± SD)		99.84 ± 1.25	
Precision (RSD)	Intraday	100.25 ± 0.98	
	Interday	101 ± 0.75	
t_R_, min	4.5 ± 0.07		
Tailing factor (T)	0.77		T ≤ 2, T = 1 for symmetric peak
Capacity factor (K’)	1.36		K’ = 1–10 acceptable
Plates number (N)	5548		N > 2000
Height equivalent to theoretical plate (HETP; cm plate^−1^)	0.04		The smaller the value, the higher the column efficiency

*Values defined by FDA Center of Drug Evaluation and Research reviewer guidance on validation of chromatographic methods (November 1994) [Online] Retrieved from www.fda.gov/downloads/Drugs/Guidances/UCM134409.pdf (accessed April 25, 2015).

### Evaluation of the removal efficiency

In this study, ZnONP and GONS were used to remove the traces of LEVO found in pharmaceutical wastewater. According to previous literature, no wastewater treatment was reported using ZnO and/or GO NPs for the removal of the antibacterial drug LEVO from wastewater. A mixed fractional factorial design (2^4^ × 3^1^) was implemented, and the studied factors (pH, exposure time, nanoparticles type and drug concentration) and their levels were chosen after preliminary trials to optimize the removal process. The studied levels for the pH factor were pH = 7 and 9; at these pH values, the LEVO has higher susceptibility to photo degradation due to the great reactivity of the ionized species existing in that range^47^. Regarding the LEVO concentration, 50 and 400 μg mL^−1^ were chosen in order to mimic the concentration range of LEVO residues found in pharmaceutical waste samples. It was found that, at LEVO concentration of 500 μg mL^−1^, the active sites ZnONP and GONS got saturated leading to a decrease in the efficiency of the drug removal percentage, while the maximum percentage achieved was ~10.23%. Subsequently, the maximum concentration of LEVO was carefully chosen to be 400 μg mL^−1^. The ratio of NPs concentration was preferred to be 5:1 (ZnONP:GONS) which will promote the photocatalytic efficiency of ZnO^48^. As obtained from the results, the ZnONP treated the wastewater samples by photocatalytic effect only; this may be elucidated due to ZnONP’s ability to absorb a great quantum of light, promoting the excitation of more electrons^49,50^. In addition, our findings revealed that GONS treated the wastewater samples by adsorption property which may be attributed to the high surface area proven by the BET analysis (about 53 m^2^ g^−1^)^51^. Thus, combining these two properties in the two different types of NPs (the photocatalytic activity of ZnONP and the high adsorption effect of GONS) will enhance the removal rate of LEVO residues.

### Analysis of the results

**Figure 8:**
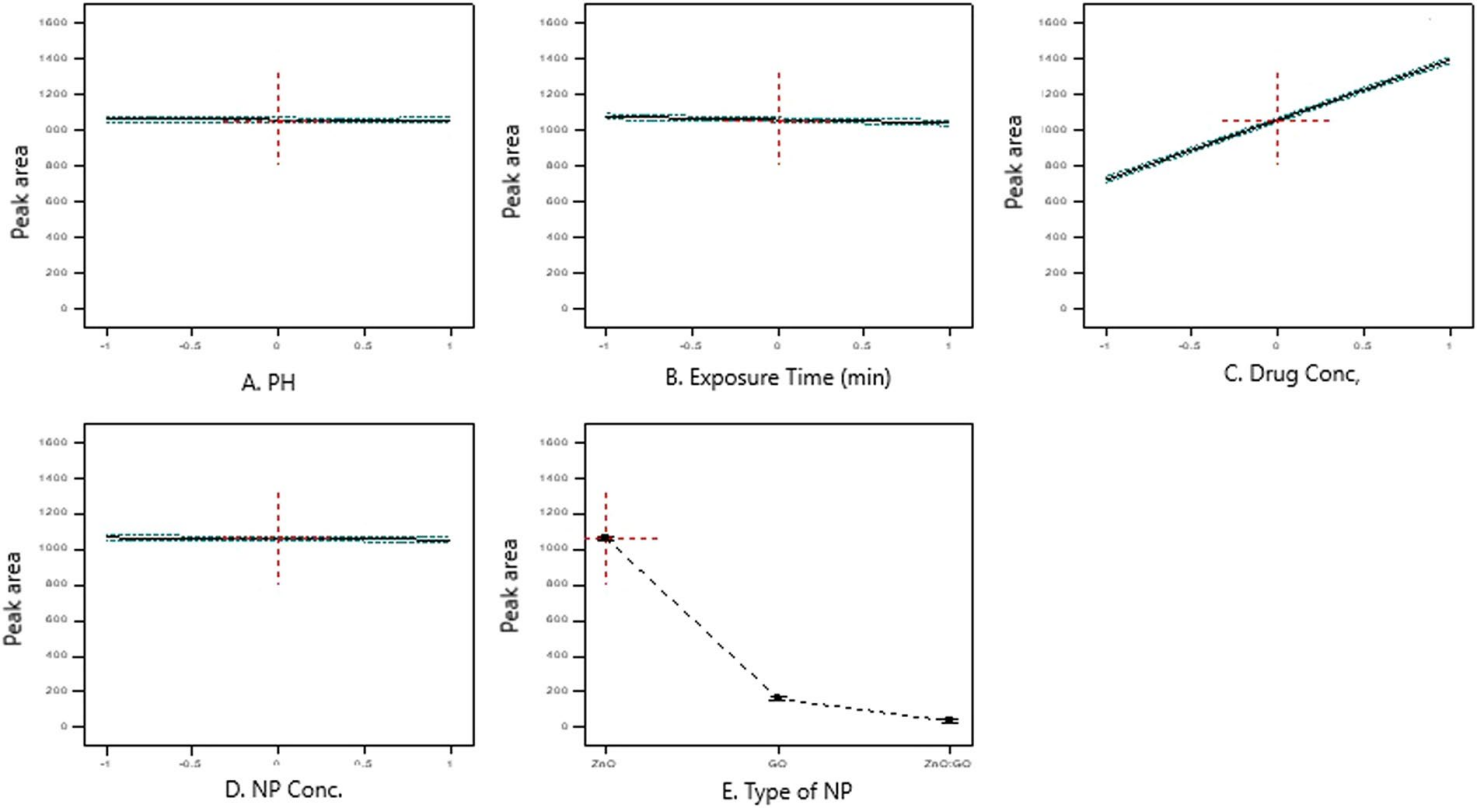
Significance of single factors on removal of LEVO.

**Figure 9:**
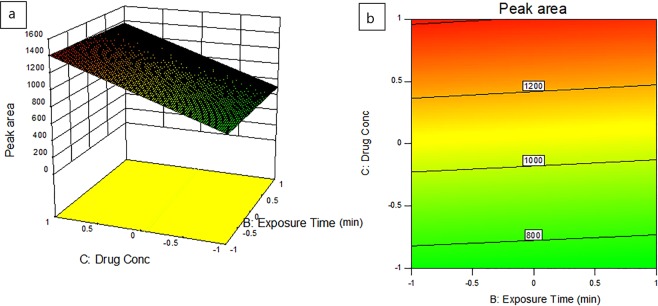
3D plot (**a**) and contour plot (**b**) of the peak area of LEVO degraded samples as a result of effect of exposure time and drug concentration.

Analysis of the mixed fractional factorial design results was carried out at a 95% confidence level using the percentage of the drug removal as the response factor. The significance of one factor has minimal effect in which R squared was 0.882, so the study of the interaction between two factors was favored and this was proven by the high R square value = 0.9966. ANOVA was carried out and the results were summarized in Table 4. The values of “Prob> F” indicated the significance of the factors if it was less than 0.05. The most significant factors on the removal efficiency were the drug concentration and the NPs types. On the other hand, the NPs concentration, the pH and the exposure time did not have an impact on the drug removal process as shown in Fig. 8. The interaction between the drug concentration (C) and nanoparticles concentration (E) sustained the highest impact on drug removal when all other factors were kept constant (Fig. 4, supplemental materials). From the results, we conclude that the usage of ZnONP alone did not significantly affect the removal process, as the maximum removal obtained was 34.12% with concentration of 1 g L^−1^. The GONS has a better effect, as 91.87% of removal was achieved; this may be because the surface area of GONS is larger than that of ZnONP. The optimum drug removal percentage obtained using the combination of both ZnONP and GONS was 99.6%. Figure 5, supplemental materials represents the normal probability plots of residual values of the LEVO treated samples. The P values (P > 0.05) indicate that the experimental points were normally distributed. The regression equation was summarized in Table 5. The 3D and contour plots show the extent of decrease in the drug concentration as a function of exposure under UV light with a constant pH, drug concentration and type of nanoparticles (ZnONP) (Fig. 9). The obtained plots agreed with the statistical ANOVA results as shown in Table 4. The performed design predicted that the most effective factors to enhance the LEVO removal (99.6%) were obtained at a lower initial concentration (50 μg mL^−1^) by means of irradiation time of 120 min and at pH = 7 using a combination of ZnONP:GONS in ratio 0.4:1 g L^−1^ (Table 6, run 35), while for 400 μg mL^−1^ LEVO initial concentration the drug removal percentage was found to be 99.21% using also a combination of ZnONP:GONS in ratio 0.4:1 g L^−1^ having the same conditions (Table 6, run 29).

**Table 4:** ANOVA for Response Surface 2FI model and analysis of variance.

Source	Sum of squares	df	Mean square	F value	p-value prob > F
Model	1.18 × 10^7−^	20	5.91 × 10^5−^	1268.36	<0.0001
A-pH	0.022	1	0.022	4.75 × 10^5−^	0.9946
B-Exposure Time	4465.5	1	4465.5	9.58	0.0045
C-Drug Conc	8.39 × 10^5−^	1	8.39 × 10^5−^	1799.19	<0.0001
D-NP Conc	830.62	1	830.62	1.78	0.1931
E-Type of NP	9.98 × 10^6−^	2	4.99 × 10^6−^	10698.35	<0.0001
AB interaction	68.31	1	68.31	0.15	0.7049
AC interaction	11.53	1	11.53	0.025	0.8762
AD interaction	927.33	1	927.33	1.99	0.1699
AE interaction	382.21	2	191.1	0.41	0.6678
BC interaction	343.76	1	343.76	0.74	0.3981
BD interaction	210.22	1	210.22	0.45	0.5076
BE interaction	2491.61	2	1245.81	2.67	0.0873
CD interaction	869.88	1	869.88	1.87	0.1832
CE interaction	9.99 × 10^5−^	2	5.00 × 10^5−^	1071.78	<0.0001
DE interaction	2351.57	2	1175.78	2.52	0.099
Residual	12587.69	27	466.21

**Table 5:** Regression Equations Summarizing the Experimental Design for LEVO.

Final Equation in Terms of Coded Factors	Peak area = 418.87 0.021 * A -9.65 * B 132.19 * C -4.16 * D 640.66 * E^1^ -257.88 * E^2^ 1.19 * AB -0.49 * AC 4.4 * AD -3.98 * AE^1^ 2.23 * AE^2^ -2.68 * BC 2.09 * BD -7.29 * BE^1^ 9.81 * BE^2^ -4.26 * CD 203.05 * CE^1^ -83.95 * CE^2^ -1.96 * DE^1^ -7.42 * DE^2^
Final Equation in Terms of Actual Factors	Peak area (**ZnO:GO)** = 36.09016 1.77765* pH -12.15985* Exposure Time 13.09875* Drug Conc 5.22234* NP Conc 1.19297* pH * Exposure Time -0.49016* pH * Drug Conc 4.39537* pH * NP Conc -2.67612* Exposure Time * Drug Conc 2.09275* Exposure Time * NP Conc -4.25704 * Drug Conc * NP Conc

**Table 6:** Solutions for the three combinations of categorical factor levels.

Number	pH	Exposure Time	Drug Conc	NP Conc	Type of NP	Peak area	Desirability	
1	−1	1	−1	−1	ZnO:GO	2.87	1	Selected
2	−1	0.983	−0.958	−0.982	ZnO:GO	3.854	1	
3	−1	0.965	−0.981	−0.967	ZnO:GO	3.847	1	

### Application to pharmaceutical wastewater samples

According to the pharmaceutical manufacturer’s protocol, the cleaning procedure was done using boiling distilled water for three successive cycles. The concentration of LEVO in the collected samples of cleaning lines was found to be around 250 μg mL^−1^ according to RP-HPLC/UV method of analysis, while the optimum conditions were applied as described above on this collected samples. After treatment using the designed NPs, the percentages of removal were 98.84 ± 0.02% and 99.17 ± 0.36% for the pharmaceutical wastewater and control samples, respectively. No significant difference in the drug removal percentage was detected which indicates the efficacy of the developed protocol and absence of matrix interference.

### Evaluation of antibacterial activity

The agar diffusion method was carried out to confirm the effective degradation of LEVO using the standard strain *Escherichia coli* ATCC 25922. The effect of adsorption and photocatalytic removal of LEVO by the combined mix of ZnONP and GONS was determined by measuring the diameter of the *Escherichia coli* inhibition zone formed. As shown in the results (Fig. 6, supplemental materials), no inhibition zone was formed around the treated wastewater sample in the agar diffusion assay. On the other hand, a 22 mm diameter zone was detectable for the untreated wastewater sample. This indicates the lack of antimicrobial activity for treated wastewater that may be referring to the absence of the antibiotic drug in water and the effective removal of LEVO from wastewater.

## Conclusion

In summary, two nanoparticles, ZnONP and GONS, and their combined form, ZnONP/GONS, were successfully fabricated and fully characterized. They have been efficiently used for the adsorptive and photocatalytic removal of LEVO from pharmaceutical wastewater. The removal efficiency was evaluated using a mixed fractional factorial design to study the influence of various factors (such as pH, the exposure time to UV radiation, the type and concentration of the NPs and the initial concentration of the antibiotic) on the potential of removal process. All the provided factors showed a significant effect except the pH and the exposure time. Furthermore, the highest removal rate was achieved using the combined nanostructure ZnONP/GONS; hence, it was applied on collected pharmaceutical wastewater samples containing LEVO residues (delivered from Egyptian pharmaceutical company) following the optimum condition. The treatment method was successfully applied for low and high antibiotic concentration levels. Subsequently, it proved to be an efficient material utilized for the removal of other antibiotics from aquatic water.

## Supplementary information

Supplementary information.

Supplementary information2.

Supplementary information3.

Supplementary information4.

Supplementary information5.

Supplementary information6.

Supplementary information7.
