# Supplementary material for: Effective Removal of Levofloxacin from Pharmaceutical Wastewater Using Synthesized Zinc Oxid, Graphen Oxid Nanoparticles Compared with their Combination
Source: Sci Rep. 2020 Apr 3;10:5914. doi: 10.1038/s41598-020-61742-4 (PMC7125086; doi:10.1038/s41598-020-61742-4)
Supplement: Supplementary file 1 — Supplementary information. [file 41598_2020_61742_MOESM1_ESM.docx]

**Effective Removal of Levofloxacin from Pharmaceutical Wastewater Using Synthesized Zinc Oxid, Graphen Oxid Nanoparticles Compared with their Combination**

Christine M. El-Maraghy ^a^, Omnia A. El-Naem ^a*^, Ola M. El-Borady ^b^

^a^Analytical Chemistry Department, Faculty of Pharmacy, October University for Modern Sciences and Arts (MSA),11787 6th October City, Egypt

^b^ Institute of Nanoscience and Nanotechnology, Kafrelsheikh University, Kafrelsheikh 33516, Egypt,

^*^ [dr.omniali@gmail.com](mailto:dr.omniali@gmail.com)

**Figure 1. Chemical structure of Levofloxacin (LEVO)**

**Figure 2. The BET image for ZnONP**

**Figure 3. The BET image for GONS**

**Figure 4. Interaction between drug concentration and nanoparticles type. The factors; PH, exposure time and nanoparticles concentration are kept constant**

**Figure 5.** **Normal probability plot of residuals for LEVO treated samples**

**Figure 6. Representative model for the antibiotic activity test using agar diffusion method for water contaminated by levofloxcine befor tretment with nanoparticles and after the treatment**
