# Supplementary figures and images for: Effective Removal of Levofloxacin from Pharmaceutical Wastewater Using Synthesized Zinc Oxid, Graphen Oxid Nanoparticles Compared with their Combination
Source: Sci Rep. 2020 Apr 3;10:5914. doi: 10.1038/s41598-020-61742-4 (PMC7125086; doi:10.1038/s41598-020-61742-4)

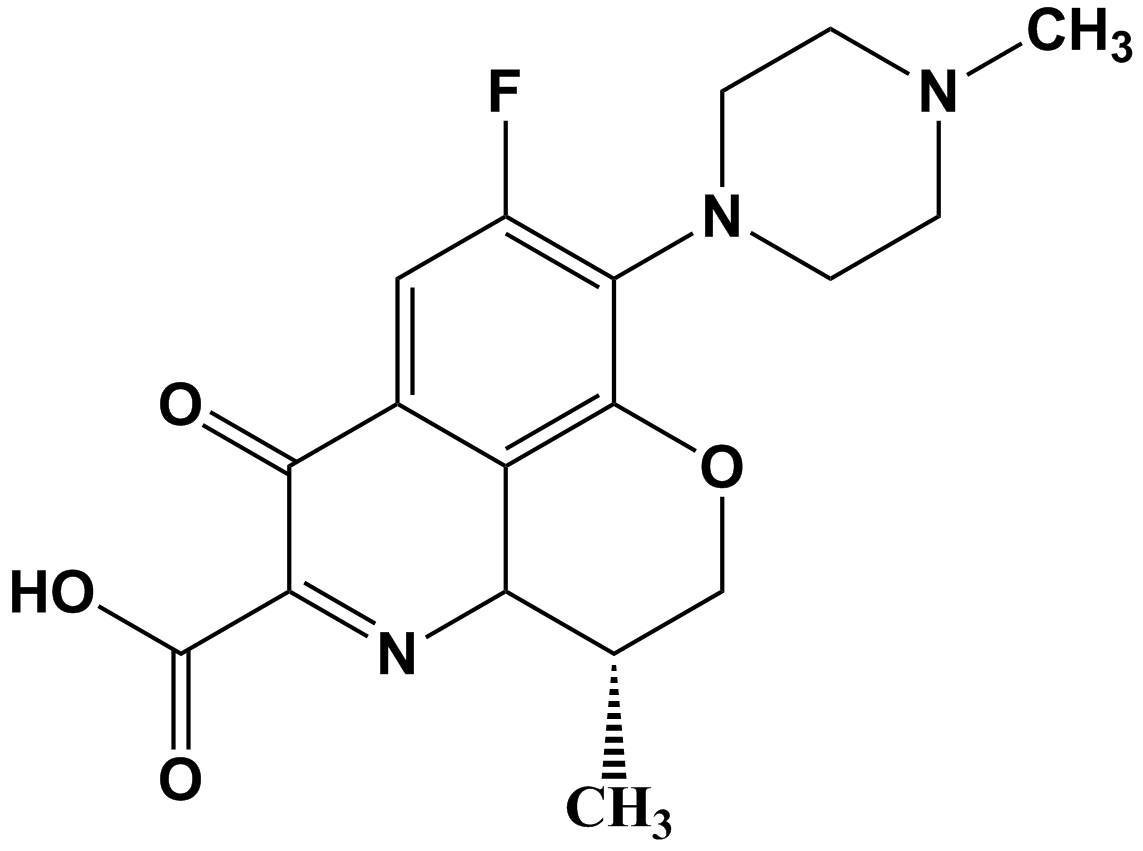

Supplement: Supplementary file 2 — Supplementary information2. [file 41598_2020_61742_MOESM2_ESM.jpg]

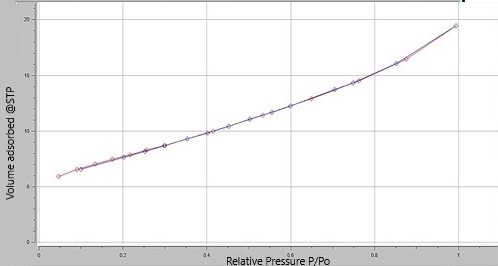

Supplement: Supplementary file 3 — Supplementary information3. [file 41598_2020_61742_MOESM3_ESM.jpg]

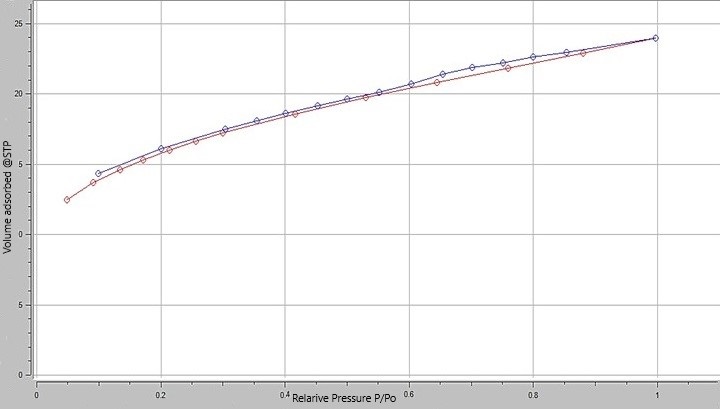

Supplement: Supplementary file 4 — Supplementary information4. [file 41598_2020_61742_MOESM4_ESM.jpg]

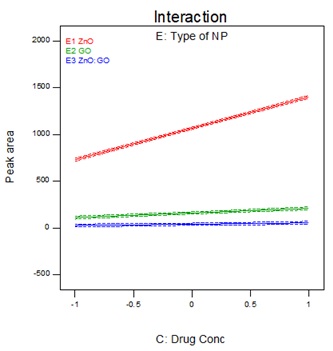

Supplement: Supplementary file 5 — Supplementary information5. [file 41598_2020_61742_MOESM5_ESM.jpg]

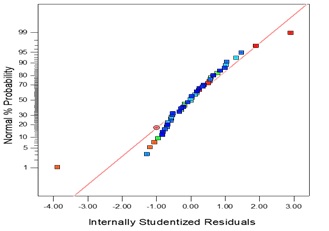

Supplement: Supplementary file 6 — Supplementary information6. [file 41598_2020_61742_MOESM6_ESM.jpg]

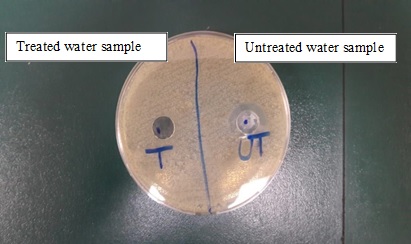

Supplement: Supplementary file 7 — Supplementary information7. [file 41598_2020_61742_MOESM7_ESM.jpg]
